# A Morphometric Study of the Mandibular Foramen, Lingula, and the Incidence of Accessory Mandibular Foramina in Dry Mandibles

**DOI:** 10.7759/cureus.81087

**Published:** 2025-03-24

**Authors:** Yashaswi Singh, Pratibha Shakya, Noor Us Saba, Heena Singh, Navneet Kumar

**Affiliations:** 1 Medicine, King George's Medical University, Lucknow, IND; 2 Anatomy, King George's Medical University, Lucknow, IND

**Keywords:** accessory foramens, lingula, mandible, mandibular foramen, reference points

## Abstract

Introduction: The foramen situated on the ramus of the mandible on its medial surface is termed the mandibular foramen (MF), which opens up into a canal. The MF acts as a conduit to the inferior alveolar nerve and vessels. Determining the location of MF is very crucial to avoid injury to the inferior alveolar nerve during inferior alveolar nerve block or surgical intervention. The lingula is a tongue-shaped projection on the medial aspect of the mandible. It is important to study the variable shapes of the lingula for surgical procedures due to its relation to the neurovascular structure. The accessory MFs are foramina other than mandibular, mental, and lingual foramina. The present study aims to locate the MF, assess the shape of the lingula, and report the incidence of accessory MFs bilaterally.

Methods: The present study was performed in the Department of Anatomy, King George's Medical University, India, on adult human mandibles of North Indian origin and unknown sex. Exclusion of mandibles was done based on deformed bones, broken bones, or any surgical procedures such as partial mandibulectomy or mandibular reconstruction affecting bony reference points for measurement of various distances from MF. After the exclusion of such types of mandibles, a total of 30 mandibles (100%) were included in the present study. Distance of MF was taken in millimeters (mm) from various reference points of the mandible (closest point on anterior and posterior border of the ramus, most distant point on angle of mandible, lowermost point of mandibular notch, highest point of coronoid process (CP)). We noted the prevalence of the shape of the lingula and the incidence of accessory MFs in percentage. A paired t-test was used as a statistical test.

Results: The mean distance of MF from the closest point of the anterior border of the ramus on the right and left sides was 17.14 ± 1.64 mm and 17.06 ± 2.18 mm, respectively (p = 0.805). The mean distance of MF from the closest point of the posterior border of the ramus on the right and left sides was 10.80 ± 1.77 mm and 10.83 ± 2.15 mm, respectively (p = 0.936). The mean distance of MF from the most distant point on the angle of the mandible on the right and left sides was 22.28 ± 3.50 mm and 22.06 ± 3.62 mm, respectively (p = 0.433). The MF was located 22.60 ± 4.20 mm and 22.53 ± 2.85 mm from the lowermost point of the mandibular notch on the right and left sides, respectively (p = 0.879). The MF was located 37.33 ± 3.62 mm and 37.42 ± 3.85 mm from the highest point of CP on the right and left sides, respectively (p = 0.803). Results revealed no significant difference in the location of MF from different bony features bilaterally. The most prevalent shape of the lingula on both sides of the mandible was truncated. Accessory MFs were present in 36.66% of mandibles.

Conclusions: The present study will help in assessing the precise location of MF, shapes of lingula, and incidence of accessory MFs, which will aid maxillofacial surgeons and oncologists in improving surgical outcomes by reducing failure of inferior alveolar nerve block. The findings for the laterality-wise location of MF were not statistically significant in the current study. For the exact localization of MF in the North Indian population, one additional criterion, i.e., distance from MF to the tip of CP, was taken in the present study.

## Introduction

The mandibular foramen (MF) leads into the mandibular canal. It is in a continuum with the roots of the molar tooth via small openings. The canal opens anteriorly on the body of the mandible as a mental foramen [1]. The MF acts as a conduit to the inferior alveolar nerve and vessels into the mandibular canal [1]. The inferior alveolar nerve innervates mandibular teeth and exits from the mandible via the mental foramen [2].

Unfortunately, there is no accurate mandibular bony feature for precise positioning of the MF [2]. Along with it, there is also a lot of variability in the morphometry of the mandibular ramus as well as the site of the MF. All these elements play a part in unsuccessful inferior alveolar neuronal blockage. It is calculated that in 20-25% of cases, inferior alveolar nerve blocks don’t become successful due to all these elements [3].

The location of the MF is also important for mandibular surgeries like vertical ramus osteotomy and inverted L osteotomy as well as aesthetic surgeries for dentofacial deformities. The inferior alveolar nerve is at increased risk during these surgical procedures. It is emphasized that awareness of the accurate position of the MF would be of immense assistance in executing sagittal split ramus osteotomy [4,5].

The lingula is a tongue-shaped projection on the medial aspect of the mandible to which the sphenomandibular ligament is attached [6]. Inferior alveolar nerves and vessels enter at the lower margin of the lingula [7]. There are various shapes of lingula - triangular, truncated, nodular, assimilated, and mixed [7]. It forms a definitive bony landmark while doing sagittal split ramus osteotomy and during inferior alveolar nerve block [6]. Almost 10-15% of failure rates of conventional nerve blocks are attributed to the structural variations of the lingula [6].

Accessory branches of the inferior alveolar nerve course through the accessory MF [5]. How far away the accessory MF is from the MF is very significant, as the effectiveness of the inferior alveolar nerve block is impacted by the diffusion of local anesthesia [8]. If there is an ineffective inferior alveolar nerve block, it may lead to painful dental procedures [8].

The smaller size of the accessory MF makes it difficult to localize in mandibular radiographic images. Panoramic imaging of the mandible also includes constraints like overlapping and deformation, which may lead to misinterpretation of key anatomic features [9].

There are only a few studies done on the North Indian population [10-13]. To the best of my knowledge, only in our study on the North Indian population did we measure the distance from the MF to the tip of the coronoid process (CP) along with other parameters for the precise location of the MF, which makes our study unique and more relevant. Our study will also substantiate the data for the exact position of the MF, lingular shape, and incidence of accessory mandibular foramina in the North Indian population.

The present study aims to locate the MF from different bony landmarks on the mandible, assess the shape of the lingula, and calculate the incidence of accessory MF in the mandible bilaterally.

## Materials and methods

The present study (August 2023 to January 2024) was carried out on adult human mandibles bilaterally of unknown sex in the Anatomy Department, King George's Medical University, Lucknow, India. A total of 30 adult human mandibles were taken for study. Ethical approval was taken from the Institutional Ethics Committee, King George's Medical University, before conducting the research (no: 1046/Ethics/2023; dated August 5, 2023).

Participant selection

Inclusion Criteria

Dry mandibles having a socket for the third molar tooth were included, as the presence of a socket for the third molar teeth is considered an indicator of an adult mandible.

Exclusion Criteria

Deformed and broken bones, any pathology of the mandible affecting its morphometry, and bones on which any surgical procedure, such as partial mandibulectomy or mandibular reconstruction, was performed, affecting bony reference points for measuring various distances from the MF, were excluded.

After the exclusion of such types of mandibles, a total of 30 mandibles (100%) were included in the present study.

Intervention and variables

A) Distances of the MF from various bony reference points of the mandibles were taken by a vernier caliper on both sides of the mandible. To remove intra-observer and inter-observer bias, each reading is taken two times by three different observers; the mean of these readings is taken for a single reading. To remove instrumental bias, the same instrument is used by all three observers each time. The tests, such as the technical error of measurement (TEM) and relative technical error of measurement (rTEM), were used for the intra-observer reliability of the measurements. For assessing interobserver reliability, the overall mean difference was calculated as per all the parameters. These methods are used for evaluating the precision and consistency of anthropometric and anatomical measurements.

**Figure 1:**
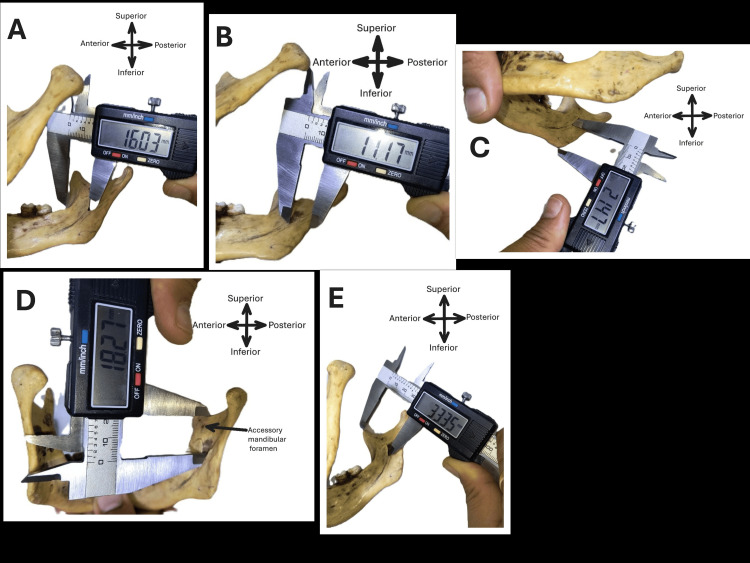
Measurement taken for the position of the mandibular foramen from various bony reference points on the mandible (mm) and the location of the accessory mandibular foramen. A: From the middle point of the anterior border of the foramen to the closest point of the anterior border of the ramus (MF-AB); B: From the middle point of the posterior border of the foramen to the closest point of the posterior border of the ramus (MF-PB); C: From the foramen to the most distant point on the angle of the mandible (MF-AG); D: From the foramen to the lowermost point of the notch of the mandible (MF-MN) and accessory mandibular foramen; E: From foramen to the highest point of the coronoid process (MF-CP)

The following parameters in millimeters (mm) were used for the accurate position of the MF (Figure 1):

1) MF-AB: From the middle point of the anterior border of the foramen to the closest point of the anterior border of the ramus

2) MF-PB: From the middle point of the posterior border of the foramen to the closest point of the posterior border of the ramus

3) MF-AG: From the foramen to the most distant point on the angle of the mandible

4) MF-MN: From the foramen to the lowermost point of the notch of the mandible

5) MF-CP: From the foramen to the highest point of the CP

B) Assessment of the shape of the lingula [7] will be done by observation. The shape of the lingula is validated by three different observers. It will be reported in percentage.

The shape was classified into triangular (wide base and pointed tip), truncated (quadrangular tip), nodular (entire lingula is merging with ramus except tip), assimilated (entire lingula is completely fused with ramus), and mixed (variable size and shape of lingula having more than one tip) [7].

C) Accessory mandibular foramina (other than mandibular, mental, and lingual foramina and the socket of teeth) were counted and reported [5]. (Figure 1D). The incidence of accessory mandibular foramina is mentioned in percentage.

Data was stored in an Excel sheet and statistically analyzed.

Sample size calculation

The minimum sample size was calculated to be 25 mandibles.

\[ N \geq \left( \frac{2 (Z_{1-\alpha/2} + Z_{1-\beta})^2}{\left(\frac{\delta_{\text{Difference}}}{\sigma_{\text{Difference}}}\right)^2} \right) + \frac{Z_{1-\alpha/2}^2}{2} \]

where alpha (α) represents the type 1 error rate; beta (β) denotes the type 2 error rate. The mean of difference (δ_Diff_) refers to the expected mean difference in the outcome between the two groups. The standard deviation of difference (σ_Diff_) indicates the expected standard deviation of the difference in the outcome between the two groups.

For the present study, alpha (α) is set at 0.05 with Z_1-α/2_ = 1.96; beta (β) is 0.2 with Z_1-β_ = 0.84; the mean of difference (δ_Diff_) is 0.5; and the standard deviation of difference (σ_Diff_) is 0.6.

The sample size is given by:

\[ N \geq \left( \frac{2 (1.96 + 0.84)^2}{\left(\frac{0.5}{0.6}\right)^2} \right) + \frac{1.96^2}{2} \]

\[ N \geq \left( 2 \times \frac{7.84}{0.694} \right) + \frac{1.96^2}{2} \]

\[ N \geq 22.59 + 1.92 \]

\[ N \geq 24.51 \approx 25 \]

The calculated minimum paired sample needed for the present study is 25.

Data management and analysis

All the data was collected and tabulated and then statistically analyzed by using the software IBM SPSS Statistics for Windows, Version 29 (Released 2021; IBM Corp., Armonk, New York, United States). Descriptive and bivariate statistical analyses were performed to fulfill the objectives of the proposed study. For continuous variables, a paired t-test was used to check the mean difference in outcome variables. A p-value less than 0.05 will be considered significant.

## Results

Absolute TEM, the variable average value (VAV), and rTEM of all the parameters were calculated (Table 1) according to Perini et al. [14]. rTEM values of all the parameters according to all observers were very low, so the reliability of all measurements was very high. All rTEM values were within acceptable limits, i.e., 10% [14].

**Table 1:** Absolute technical error of measurement (TEM) and relative technical error of measurement (rTEM) values of all the parameters for measurement reliability MF-AB: from the middle point of the anterior border of the foramen to the closest point of the anterior border of the ramus; MF-PB: from the middle point of the posterior border of the foramen to the closest point of the posterior border of the ramus; MF-AG: from the foramen to the most distant point on the angle of the mandible; MF-MN: from the foramen to the lowermost point of the notch of the mandible; MF-CP: from the foramen to the highest point of the coronoid process; VAV: variable average value; absolute TEM: absolute technical error of measurement; rTEM: relative technical error of measurement

Parameters for measurement	Observer 1				Observer 2				Observer 3			
	Mean ± SD (mm)	Absolute TEM %	VAV (mm)	Relative TEM %	Mean ± SD (mm)	Absolute TEM %	VAV (mm)	Relative TEM %	Mean ± SD (mm)	Absolute TEM %	VAV (mm)	Relative TEM %
Right MF-AB	17.14 ± 1.64	0.03	17.14	0.18	17.13 ± 1.64	0.03	17.14	0.18	17.13 ± 1.64	0.03	17.14	0.18
	17.13 ± 1.65				17.15 ± 1.65				17.15 ± 1.65			
Left MF-AB	17.06 ± 2.18	0.04	17.07	0.21	17.07 ± 2.18	0.03	17.06	0.20	17.06 ± 2.18	0.03	17.06	0.18
	17.08 ± 2.18				17.06 ± 2.18				17.06 ± 2.17			
Right MF-PB	10.80 ± 1.77	0.04	10.80	0.37	10.79 ± 1.77	0.04	10.80	0.37	10.80 ± 1.77	0.03	10.80	0.28
	10.80 ± 1.77				10.81 ± 1.78				10.81 ± 1.78			
Left MF-PB	10.83 ± 2.15	0.04	10.83	0.37	10.83 ± 2.15	0.03	10.83	0.28	10.82 ± 2.15	0.03	10.83	0.28
	10.82 ± 2.15				10.83 ± 2.15				10.84 ± 2.15			
Right MF-AG	22.27 ± 3.50	0.03	22.28	0.13	22.28 ± 3.50	0.04	22.28	0.18	22.28 ± 3.50	0.04	22.28	0.18
	22.29 ± 3.50				22.29 ± 3.50				22.29 ± 3.49			
Left MF-AG	22.06 ± 3.61	0.03	22.06	0.14	22.06 ± 3.63	0.03	22.06	0.14	22.07 ± 3.62	0.03	22.06	O.14
	22.07 ± 3.64				22.06 ± 3.62				22.05 ± 3.63			
Right MF-MN	22.59 ± 4.20	0.03	22.60	0.13	22.58 ± 4.20	0.04	22.60	0.18	22.59 ± 4.19	0.03	22.60	0.13
	22.61 ± 4.19				22.61 ± 4.19				22.61 ± 4.20			
Left MF-MN	22.53 ± 2.86	0.03	22.53	0.13	22.53 ± 2.84	0.04	22.53	0.18	22.53 ± 2.86	0.04	22.53	0.18
	22.53 ± 2.84				22.53 ± 2.85				22.53 ± 2.85			
Right MF-CP	37.32 ± 3.62	0.05	37.33	0.13	37.33 ± 3.62	0.05	37.33	0.13	37.33 ± 3.61	0.06	37.34	0.16
	37.33 ± 3.62				37.34 ± 3.62				37.35 ± 3.61			
Left MF-CP	37.41 ± 3.86	0.05	37.43	0.13	37.41 ± 3.87	0.05	37.42	0.13	37.42 ± 3.86	0.05	37.43	0.13
	37.44 ± 3.84				37.43 ± 3.85				37.44 ± 3.85			

For assessing interobserver reliability, the overall mean difference was calculated for all parameters. The overall mean difference for right MF-AB and left MF-AB was 0.02 mm each; for right MF-PB and left MF-PB, it was 0.02 mm each; for right MF-AG and left MF-AG, it was 0.02 mm each; for right MF-MN and left MF-MN, it was 0.03 mm and 0 mm, respectively; and for right MF-CP and left MF-CP, it was 0.03 mm each.

As the value of the overall mean difference was very low in all the parameters, the interobserver reliability of all measurements was very high.

In the present study, for every variable, the paired t-test revealed a p-value greater than 0.05. It signifies that the null hypothesis is accepted, i.e., there was no difference in the location of the MF when we compared the mandible bilaterally (Table 2).

**Table 2:** Distance of the mandibular foramen was taken from various bony reference points of the mandibles (mm) MF-AB: from the middle point of the anterior border of the foramen to the closest point of the anterior border of the ramus; MF-PB: from the middle point of the posterior border of the foramen to the closest point of the posterior border of the ramus; MF-AG: from the foramen to the most distant point on the angle of the mandible; MF-MN: from the foramen to the lowermost point of the notch of the mandible; MF-CP: from the foramen to the highest point of the coronoid process

	Distance of mandibular foramen was taken from various bony reference points (mm)	Mean ± SD (mm)	Mean difference	% mean change	p-value	t value
Pair 1	MF-AB right	17.14 ± 1.64	0.07	0.43	0.805	0.249
	MF-AB left	17.06 ± 2.18				
Pair 2	MF-PB right	10.80 ± 1.77	-0.03	-0.26	0.936	-0.081
	MF-PB left	10.83 ± 2.15				
Pair 3	MF-AG right	22.28 ± 3.50	0.22	0.99	0.433	0.795
	MF-AG left	22.06 ± 3.62				
Pair 4	MF-MN right	22.60 ± 4.20	0.07	0.31	0.879	0.154
	MF-MN left	22.53 ± 2.85				
Pair 5	MF-CP right	37.33 ± 3.62	-0.09	-0.24	0.803	-0.251
	MF-CP left	37.42 ± 3.85				

For MF-AB, the MF was found more distant to the anterior border of the ramus on the right side (17.14 ± 1.64 mm) compared to the left side (17.06 ± 2.18 mm), p = 0.805, t = 0.249. For MF-PB, the MF was found closer to the posterior border of the ramus on the right side (10.80 ± 1.77 mm) compared to the left side (10.83 ± 2.15 mm), p = 0.936, t = -0.081 (Table 2).

For MF-AG, the MF was found more distant to the angle of the mandible on the right side (22.28 ± 3.50 mm) compared to the left side (22.06 ± 3.62 mm), p = 0.433, t = 0.795. For MF-MN, the MF was found more distant to the mandibular notch on the right side (22.60 ± 4.20 mm) compared to the left side (22.53 ± 2.85 mm), p = 0.879, t = 0.154. For MF-CP, the MF was found closer to the highest point of the CP on the right side (37.33 ± 3.62 mm) compared to the left side (37.42 ± 3.85 mm), p = 0.803, t = -0.251 (Table 2).

**Figure 2:**
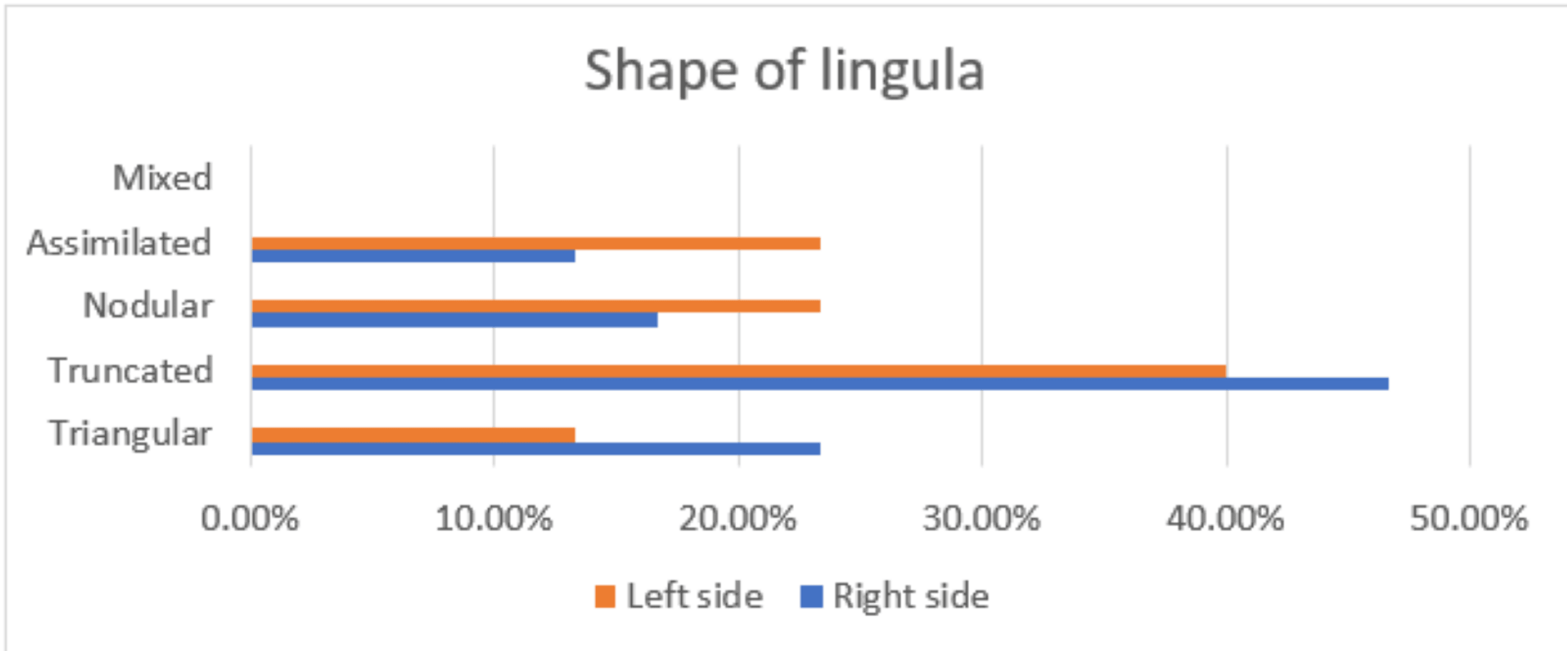
Bar graph showing the incidence of the shape of the lingula

According to our study, the most prevalent shape of the lingula on both sides of the mandible was truncated, and the least prevalent shapes of the lingula on the right side and left side were assimilated and triangular, respectively. We didn't find mixed types of lingula in the present study (Table 3, Figure 2).

**Table 3:** Shape of lingula

Shape of lingula (60 sides)	Right side (30 sides)	Left side (30 sides)
Triangular (11 sides or 18.33%)	7 sides (23.33%)	4 sides (13.33%)
Truncated (26 sides or 43.33%)	14 sides (46.67%)	12 sides (40%)
Nodular (12 sides or 20% )	5 sides (16.67%)	7 sides (23.33%)
Assimilated (11 sides or 18.33%)	4 sides (13.33%)	7 sides (23.33%)
Mixed (0 sides or 0%)	0 sides (0%)	0 sides (0%)

In the present study, accessory mandibular foramina were present in 36.67% of mandibles, mostly on the medial aspect of mandibles. Unilaterally, its incidence was 13.33% on the right side and 6.67% on the left side. Bilaterally, its incidence was 16.67% (Table 4).

**Table 4:** Incidence of accessory mandibular foramens

Accessory mandibular foramen	Number of accessory mandibular foramens	Number of mandibles (30)	Site of accessory mandibular foramens
Right side	1	4 (13.33%)	3 mandibles - on medial surface of ramus
			1 mandible - on lateral surface of ramus
Left side	1	2 (6.67%)	2 mandibles - on medial surface of ramus
Bilateral	1	4 (13.33%)	3 mandibles bilaterally - on medial surface of ramus 1 mandible - lateral surface of ramus (right side), medial surface of ramus (left side)
	Right side - 1 Left side - 2	1 (3.33%)	1 on right side of 1 mandible - lateral surface of ramus 2 on left side of same 1 mandible - medial surface of ramus
Absent	0	19 (63.33%)	0

## Discussion

In the present study, the mean distance of MF was taken from various reference points of the mandible (closest point on the anterior border of the ramus (right side: 17.14 ± 1.64 mm; left side: 17.06 ± 2.18 mm; p = 0.805), and the closest point on the posterior border of the ramus (right side: 10.80 ± 1.77 mm; left side: 10.83 ± 2.15 mm; p = 0.936), the most distant point on the angle of the mandible (right side: 22.28 ± 3.50 mm; left side: 22.06 ± 3.62 mm; p = 0.433), lowermost point of mandibular notch (right side: 22.60 ± 4.20 mm; left side: 22.53 ± 2.85 mm; p = 0.879), and highest point of CP (right side: 37.33 ± 3.62 mm; left side: 37.42 ± 3.85 mm; p = 0.803)). It revealed no significant difference in the location of the MF from different bony features bilaterally. The most prevalent shape of the lingula on both sides of the mandible was truncated. Accessory MFs were present in 36.66% of mandibles. The findings of the present study can substantiate the data present in the existing literature.

The novelty of the study is to measure the distance of MF from the highest point of the CP which was not done in other studies on the North Indian population. This is a strength and contribution to existing literature.

Location of mandibular foramen

The awareness of the location of the MF from different bony landmarks of the mandible is very important because it is impossible to palpate the foramen from inside the oral cavity or from the surface superficially.

The advancement of implant techniques has heightened interest in the anatomy of the mandible, particularly in accurately locating the MF [2].

Oguz and Bozkir [15] conducted a study on the Turkish population and concluded that the MF was located posterior to the center of the ramus of the mandible on both sides. In the present study, we also found the same finding, which also coincides with the studies done in the South and North Indian populations [2,5,10,11,16-18], mentioned in Table 5.

The following points can be suggested by the present study in the North Indian population. First, for successful inferior alveolar nerve blockage, local anesthesia should be injected posterior to the center of the ramus of the mandible. Second, if a fracture line is present posterior to the center of the ramus of the mandible, then it may injure the inferior alveolar nerve.

Gopalakrishna et al. [17] compared their study to a study done in Pakistan and different parts of India. They found no geographical difference in Pakistan's population, South Indian population, and North Indian population.

According to Khan and Ansari [10], accurate and exact awareness of the position of the MF is very crucial as it provides a successful inferior alveolar nerve block, which leads to a painless dental procedure for the patient.

All the distances of the MF from various reference points for the location of the MF in various studies are compared in Table 5.

**Table 5:** Distance of mandibular foramen was taken from various bony reference points of mandibles (mm) MF-AB: from the middle point of the anterior border of the foramen to the closest point of the anterior border of the ramus; MF-PB: from the middle point of the posterior border of the foramen to the closest point of the posterior border of the ramus; MF-AG: from the foramen to the most distant point on the angle of the mandible; MF-MN: from the foramen to the lowermost point of the notch of the mandible; MF-CP: from the foramen to the highest point of the coronoid process

Author	Population	Side	MF-AB	MF-PB	MF-AG	MF-MN	MF-CP
Oguz and Bozkir (2002) [15]	Turkey (34 mandibles)	Right	16.09 ± 2.27	14.09 ± 2.27	Not observed	22.37 ± 3.64	Not observed
		Left	16.78 ± 2.99	14.37 ± 2.56	Not observed	22.17 ± 4.41	Not observed
Samanta and Kharb (2013) [16]	Indian (60 mandibles)	Right	15.72 ± 2.92	13.29 ± 1.74	21.54 ± 2.92	22.70 ± 3.00	Not observed
		Left	16.23 ± 2.88	12.73 ± 2.04	21.13 ± 3.43	22.27 ± 2.62	Not observed
Padmavathi et al. (2014) [2]	South Indian (65 mandibles)	Right	± 2.8	11.7 ± 2.0	22.6 ± 3.4	22.0 ± 3.0	Not observed
		Left	± 2.5	12.1 ± 2.4	22.2 ± 2.9	22.3 ± 3.4	Not observed
Shalini et al. (2016) [5]	South Indian (204 mandibles)	Right	± 2.74	10.47 ± 2.11	Not observed	21.74 ± 2.74	Not observed
		Left	± 3.05	9.68 ± 2.03	Not observed	21.92 ± 3.33	Not observed
Gopalakrishna et al. (2016) [17]	South Indian (100 mandibles)	Right	± 3.16	12.34 ± 3.10	22.14 ± 3.18	Not observed	Not observed
		Left	± 3.11	13.51 ± 3.92	22.11 ± 4.12	Not observed	Not observed
Khan and Ansari (2016) [10]	North Indian (30 mandibles)	Right	± 1.99	12.02 ± 1.99	19.64 ± 4.03	18.79 ± 2.79	Not observed
		Left	16.13 ± 2.10	11.10 ± 1.95	19.49 ± 4.19	18.71 ± 2.77	Not observed
Gupta et al. (2016) [11]	North Indian (45 mandibles)	Right	18.9 ± 2.14	14.31 ± 1.82	Not observed	Not observed	Not observed
		Left	18.88 ± 2.34	14.39 ± 1.79	Not observed	Not observed	Not observed
Sultana and Sreekanth (2019) [18]	South Indian (60 mandibles)	Right	± 2.73	12.67 ± 2.37	23.00 ± 3.92	21.04 ± 2.95	35.68 ± 3.25
		Left	± 2.52	13.05 ± 2.43	22.36 ± 3.89	20.24 ± 2.94	35.19 ± 3.47
Present study	North Indian (30 mandibles)	Right	± 1.64	10.80 ± 1.77	22.28 ± 3.50	22.60 ± 4.20	37.33 ± 3.62
		Left	± 2.18	10.83 ± 2.15	22.06 ± 3.62	22.53 ± 2.85	37.42 ± 3.85

All the studies shown in Table 5 didn't conduct studies on male and female mandibles separately.

The findings of the present study (MF-AB and MF-PB) were closer to the findings of Padmavathi et al. [2] and Shalini et al. [5]. Findings regarding MF-AG of our study were closer to Gopalakrishna et al. [17] and Sultana and Sreekanth [18]. The finding of MF-MN was closer to Oguz and Bozkir [15], Samanta and Kharb [16], and Padmavathi et al. [2]. The finding regarding MF-CP of the present study was closer to Sultana and Sreekanth [18]. Sultana and Sreekanth [18] was the only study that measured MF-CP, but it was a South Indian study.

Our study’s findings may be dissimilar to the findings of other studies done in the north Indian population because of differences in sample size and differences in criteria taken for measurement of various distances.

Our study adds one more criterion, i.e., distance from the MF to the tip of the CP, for the exact localization of the MF, which was not done in the above North Indian studies.

Shape of lingula

For the first time, Tuli et al. [19] classified the lingular shape into triangular, truncated, nodular, and assimilated. Ogut and Yildirim [7] found mixed types of lingula in addition to the above, but other authors [18-26] mentioned in Table 6 didn't find any mixed type of lingula in their study, just like the present study. Hossain et al. [20] didn't find nodular and mixed lingula. All these variations may be due to geographical differences.

The most common and least common lingual shapes are discussed in other research articles in Table 6.

**Table 6:** Shape of lingula (N or %)

Author	Population and sample size	Most common shape of lingula (N or %)		Least common shape of lingula (N or %)	
Tuli et al. (2000) [19]	New Delhi (330 sides)	Triangular (226 sides or 68.5%)		Assimilated (16 sides or 4.8%)	
Hossain et al. (2001) [20]	Bangladeshi (416 sides)	Triangular (292 sides or 70.2%)		Assimilated (40 sides or 9.6%)	
Devi et al. (2003) [21]	South Indian (294 sides)	Truncated and nodular		Assimilated	
Kositbowornchai et al. (2007) [22]	Thailand (144 sides)	Truncated (68 sides or 47%)		Assimilated (18 sides or 13%)	
Jansisyanont et al. (2009) [23]	Thailand (146 sides)	Truncated (67 sides or 46.2%)		Assimilated (6 sides or 4.3%)	
Lopes et al. (2010) [24]	South Brazil (160 sides)	Triangular (66 sides or 41.3%)		Assimilated (19 sides or 11.9%)	
Nirmale et al. (2012) [25]	Indian (168 sides)	Triangular (80 sides or 47.61%)		Truncated (18 sides or 10.71%)	
Padmavathi et al. (2014) [26]	South Indian (130 sides)	Truncated (44 sides or 33.84%)		Assimilated (23 sides or 17.70%)	
Asdullah et al. (2018) [12]	North Indian (100 sides)	Triangular (62 sides or 62%)		Assimilated (12 sides or 12%)	
Ogut and Yildirim (2021) [7]	Turkish (200 sides)	Truncated (84 sides or 42%)		Mixed means having more than 1 tip (3 sides or 1.5%)	
Present study	North Indian (60 sides)	Right	Left	Right	Left
		Truncated	Truncated	Assimilated	Triangular
		46.67%	40%	13.33%	13.33%

Tuli et al. [19] found no dissimilarity regarding the most common and less common shapes of lingula between male and female mandibles. Hossain et al. [20] and Nirmale et al. [25] found more incidence of each type of shape of lingula in the mandibles of males in comparison to females. Asdullah et al. [12] found the same incidence of truncated and assimilated in both males and females, but they found fewer triangular and more nodular in males in comparison to females.

Ogut and Yildirim [7], Devi et al. [21], Kositbowornchai et al. [22], Jansisyanont et al. [23], Lopes et al. [24], and Padmavathi et al. [26] didn't conduct a gender-wise study regarding the shape of the lingula.

In our present study, we found truncated as the most common variant but assimilated on the right side and triangular on the left side of the mandible as the least common variant, which was closer to the study done by Kositbowornchai et al. [22] and Padmavathi et al. [26].

In the present study, there was a distinction in the shape of the lingula between the right and left sides as the least common variant. We found it assimilated on the right side (13.33%) and triangular on the left side (13.33%). Similarly, Lopes et al. [24] found assimilated-shaped lingula as the least common variant on the right side (10%) and nodular-shaped lingula as the least common variant on the left side (10%). They found triangular-shaped lingula as the most common variant on both the right side (42.50%) and the left side (40%).

All the studies mentioned in Table 6 show variations in the incidence of the most common and less common shapes of lingula due to geographical differences.

Studies conducted on North India or South India in Table 6 also do not represent the whole of North India or South India, but they represent only a small area of that region. Due to this, all North Indian studies or all South Indian studies do not show similar incidence of the most common and less common shape of lingula.

Incidence of accessory mandibular foramens 

The incidence of accessory MFs is compared with other studies in Table 7.

**Table 7:** Incidence of accessory mandibular foramens (N or %)

Author	Population	Location of accessory mandibular foramens				Percentage of accessory mandibular foramens
Freire et al. (2012) [27]	Brazilian (222 mandibles)	Unilateral			Bilateral	(158 mandibles or 71.16%)
		(105 mandibles or 47.29%)			(53 mandibles or 23.87%)	
Samanta and Samanta (2013) [16]	Indian (60 mandibles)	Unilateral			Bilateral	(10 mandibles or 16.66%)
		(6 mandibles or 10%)			(4 mandibles or 6.66%)	
Padmavathi et al. (2014) [2]	South Indian (65 mandibles)	Unilateral			Bilateral	(27 mandibles or 41.5%)
		Right		Left		
		(8 mandibles or 12.3%)		(11 mandibles or 16.9%)	(8 mandibles or 12.3%)	
Gopalakrishna et al. (2016) [17]	South Indian (100 mandibles)	Not classified as unilateral or bilateral				(18 mandibles or 18%)
Shalini et al. (2016) [5]	South Indian (204 mandibles)	Unilateral			Bilateral	(66 mandibles or 32.36%)
		Right		Left	(21 mandibles or 10.3%)	
		(25 mandibles or 12.25%)		(20 mandibles or 9.81%)		
Asdullah et al. (2018) [12]	North Indian (50 mandibles)	Right			Left	(14 mandibles or 28%)
		(6 mandibles or 12%)			(8 mandibles or 16%)	
Singh et al. (2020) [13]	North Indian (28 mandibles)	Unilateral			Bilateral	(11 mandibles or 39.29%)
		(5 mandibles or 17.86%			(6 mandibles or 21.43%)	
Present study	North Indian (30 mandibles)	Unilateral			Bilateral	(11 mandibles or 36.67%)
		Right	Left		(5 mandibles or 16.67%)	
		(4 mandibles or 13.33%)	(2 mandibles or 6.67%)			

Only Asdullah et al. [12] in Table 7 conducted a gender-wise study regarding the incidence of accessory mandibular foramina and found more incidence in females in comparison to males. Other authors [2,5,13,16,17,27] didn't conduct a genderwise study regarding the incidence of accessory mandibular foramina.

The incidence of accessory mandibular foramina in our study was closer to the study done by Shalini et al. [5] and Singh et al. [13].

Chávez-Lomeli et al. described that initially in the embryonic period, inferior alveolar nerves were three in number and led to the formation of additional nutrient canals or foramina. Subsequently, these nerves unite to form one inferior alveolar nerve. Variability in nerve branching during ossification can result in accessory mandibular foramina, which persist into adulthood [28].

The awareness of accessory mandibular foramina in planning dental procedures and surgeries such as mandibular osteotomies and implant placement is very important. It helps in making the inferior alveolar nerve block successful. Accessory mandibular foramina provide a route for the spread of cancerous cells, so knowledge of these foramina is very necessary to plan radiation treatment [17].

Sakalem et al. (2024) conducted a study on 63 dried mandibles in the Department of Anatomy of the State University of Londrina and found 6.35% accessory mandibular foramina. They said that unawareness of the presence of accessory mandibular foramina may damage accessory blood vessels and nerve branches, which may lead to intraoperative excessive bleeding and postoperative nerve dysfunction [29].

Understanding the positions of accessory mental foramina in the mandible is also crucial because it may impact surgical outcomes [30].

All the studies mentioned in Table 6 show variations in the incidence of accessory mandibular foramina due to geographical differences. Studies mentioned in North India or South India in Table 7 also do not represent the whole of North India or South India, but they represent only a small area of that region. Due to this, all North Indian studies or all South Indian studies do not show similar incidences of accessory mandibular foramina.

Future prospects of study

Imaging-based validation, such as the use of cone-beam computed tomography (CBCT) or CT scans and 3D analysis, should be considered in future studies to increase sample size and more accurate measurements. The morphometric method to define the shape of the lingula can also increase the reliability of the study. A more diverse sample, including mandibles from other regions or ethnic groups, should be taken in future studies to strengthen the generalizability of the findings.

Limitations of study

A few limitations have been observed in the study. First, the sample size was less to determine the significant statistical test. Second, gender distribution was not performed due to already procured dry mandibles of unknown sex. Third, age-wise distribution was not done as previous data on the exact age of these procured mandibles was not available. Fourth, a limited geographic sample (mandibles of North Indian origin) may not fully represent the broader population's anatomical diversity.

## Conclusions

To achieve precise localization of the MF, an additional parameter - the distance from the MF to the tip of the CP - can be helpful. The current study offers a reasonable understanding of the location of the MF, the shape of the lingula, and accessory MF, which will be helpful for maxillofacial surgeons, radiologists, and oncologists for performing surgeries on the mandible. Additionally, the data can be helpful for anthropological evaluations and reconstructive surgical procedures.

## References

[1] Kilarkaje N, Nayak SR, Narayan P (2005). The location of the mandibular foramen maintains absolute bilateral symmetry in mandibles of different age groups. Hong Kong Dent J.

[2] Padmavathi G, Tiwari S, Varalakshmi KL (2014). An anatomical study of mandibular and accessory mandibular foramen in dry adult human mandibles of South Indian origin. IOSR-JDMS.

[3] Quinn JH (1998). Inferior alveolar nerve block using the internal oblique ridge. J Am Dent Assoc.

[4] Daw JL Jr, de la Paz MG, Han H, Aitken ME, Patel PK (1999). The mandibular foramen: an anatomic study and its relevance to the sagittal ramus osteotomy. J Craniofac Surg.

[5] Shalini R, RaviVarman C, Manoranjitham R, Veeramuthu M (2016). Morphometric study on mandibular foramen and incidence of accessory mandibular foramen in mandibles of south Indian population and its clinical implications in inferior alveolar nerve block. Anat Cell Biol.

[6] Senel B, Ozkan A, Altug HA (2015). Morphological evaluation of the mandibular lingula using cone-beam computed tomography. Folia Morphol (Warsz).

[7] Ogut E, Yildirim FB (2021). The effects of relationship between the mixed typed of lingula and coronoid process of the mandible. J DEU Med.

[8] Parirokh M, Yosefi MH, Nakhaee N, Abbott PV, Manochehrifar H (2015). The success rate of bupivacaine and lidocaine as anesthetic agents in inferior alveolar nerve block in teeth with irreversible pulpitis without spontaneous pain. Restor Dent Endod.

[9] Pancer B, Garaicoa-Pazmiño C, Bashutski JD (2014). Accessory mandibular foramen during dental implant placement: case report and review of literature. Implant Dent.

[10] Khan IA, Ansari MA (2016). An anatomical study and clinical correlations of mandibular foramen in dry adult human mandibles of North Indian origin. Ann Int Med Den Res.

[11] Gupta P, Bharati N, Hussein M (2016). Clinical implications of variations in the position of mandibular foramen in North Indian mandibles. J Anat Soc India.

[12] Asdullah M, Ansari AA, Khan MH, Salati NA, Khawja KJ, Sachdev AS (2018). Morphological variations of lingula and prevalence of accessory mandibular foramina in mandibles: a study. Natl J Maxillofac Surg.

[13] Singh A, Hasan S, Zaidi H (2020). A study of accessory mandibular foramina in north Indian mandibles. Int J Recent Trends Sci Technol.

[14] Perini TA, Oliveira GLD, Ornellas JDS (2005). Technical error of measurement in anthropometry. Rev Bras Med Esporte.

[15] Oguz O, Bozkir MG (2002). Evaluation of location of mandibular and mental foramina in dry young adult human male dentulous mandibles. West Indian Med J.

[16] Samanta PP, Kharb P (2013). Morphometric analysis of mandibular foramen and incidence of accessory mandibular foramen in adult human mandibles of an Indian population. Rev Arg De Anat Clin.

[17] Gopalakrishna K, Deepalaxmi S, Somashekara SC, Rathna BS (2016). An anatomical study on the position of mandibular foramen in 100 dry mandibles. Int J Anat Res.

[18] Sultana Z, Sreekanth T (2019). A morphometric study of mandibular foramen in dry adult human mandibles of Indian population in Telangana state. Int J Anat Res.

[19] Tuli A, Choudhry R, Choudhry S, Raheja S, Agarwal S (2000). Variation in shape of the lingula in the adult human mandible. J Anat.

[20] Hossain SMA, Patwary SI, Karim M (2001). Variation in shape of the lingulae in the adult human mandibles of Bangladeshi skulls. Pak J Med Sci.

[21] Devi R, Arna N, Manjunath KY, Balasubramanyam Balasubramanyam (2003). Incidence of morphological variants of mandibular lingula. Indian J Dent Res.

[22] Kositbowornchai S, Siritapetawee M, Damrongrungruang T, Khongkankong W, Chatrchaiwiwatana S, Khamanarong K, Chanthaooplee T (2007). Shape of the lingula and its localization by panoramic radiograph versus dry mandibular measurement. Surg Radiol Anat.

[23] Jansisyanont P, Apinhasmit W, Chompoopong S (2009). Shape, height, and location of the lingula for sagittal ramus osteotomy in Thais. Clin Anat.

[24] Lopes PTC, Pereira GAM, Santos AMPV (2010). Morphological analysis of the lingula in dry mandibles of individuals in Southern Brazil. J Morphol Sci.

[25] Nirmale VK, Mane UW, Sukre SB (2012). Morphological features of human mandible. Int J Recent Trends Sci Technol.

[26] Padmavathi G, Varalakshmi KL, Tiwari S (2014). A morphological and morphometric study of the Lingula in dry adult human mandibles of South Indian origin and its clinical significance. Int J Health Res.

[27] Freire AR, Rossi AC, Prado FB (2012). Incidence of the mandibular accessory foramina in Brazilian population. J Morphol Sci.

[28] Chávez-Lomeli ME, Mansilla Lory J, Pompa JA, Kjaer I (1996). The human mandibular canal arises from three separate canals innervating different tooth groups. J Dent Res.

[29] Sakalem ME, Sestario CS, Motta AL (2024). Anatomical variations of the human mandible and prevalence of duplicated mental and mandibular foramina in the collection of the State University of Londrina. Transl Res Anat.

[30] Sindel A, Ogut E, Kastan O (2017). Position, variation and asymmetry of the mental foramen: a morphological study. ​Eur J Ther​.

